# Possible Association Between Concomitant Use of SSRIs with NSAIDs and an Increased Risk of Adverse Events Among People with Depressive Disorders: Data Mining of FDA Adverse Event Reporting System

**DOI:** 10.3390/ph18071062

**Published:** 2025-07-18

**Authors:** Yi Zhang, Xiaoyu Liu, Jianru Wu, Xuening Zhang, Fenfang Wei, Limin Li, Hongqiao Li, Xinru Wang, Bei Wang, Wenyu Wu, Xiang Hong

**Affiliations:** 1 Key Laboratory of Environmental Medicine and Engineering of Ministry of Education, School of Public Health, Southeast University, Nanjing 210009, China; zhangyi_fly@163.com (Y.Z.);; 2 Shenzhen Institute of Pharmacovigilance and Risk Management, Shenzhen 518024, China; liuxy4@mail.amr.sz.gov.cn (X.L.);; 3 Jiangsu Health Development Research Center, Nanjing 210029, China; 4 NHC Key Laboratory of Contraceptives Vigilance and Fertility Surveillance, Nanjing 210029, China; 5 Jiangsu Provincial Medical Key Laboratory of Fertility Protection and Health Technology Assessment, Nanjing 210029, China

**Keywords:** drug–drug interactions, adverse event, Adverse Event Reporting System, FAERS database, data mining

## Abstract

**Background**: Depression, a major global health issue, is commonly treated with selective serotonin reuptake inhibitors (SSRIs). Given the link between depression and inflammation, nonsteroidal anti-inflammatory drugs (NSAIDs) may have adjunctive benefits. Clinically, SSRIs and NSAIDs are often co-prescribed for comorbid pain or inflammatory conditions. However, both drug classes pose risks of adverse effects, and their interaction may lead to clinically significant drug–drug interactions. **Objectives**: This study analyzed FDA Adverse Event Reporting System (FAERS) data (2004–2024) to assess gastrointestinal bleeding, thrombocytopenia, and acute kidney injury (AKI) potential risks linked to SSRIs (citalopram, escitalopram, fluoxetine, paroxetine, fluvoxamine, and sertraline) and NSAIDs (propionic/acetic/enolic acid derivatives, COX-2 inhibitors) in depression patients, alone and combined. **Methods**: Disproportionality analysis (crude reporting odds ratios, cROR) identified possible associations; drug interactions were evaluated using Ω shrinkage, additive, multiplicative, and combination risk ratio (CRR) models. **Results**: Gastrointestinal bleeding risk was potentially elevated with citalopram (cROR = 2.81), escitalopram (2.27), paroxetine (2.17), fluvoxamine (3.58), sertraline (1.69), and propionic acid NSAIDs (3.17). Thrombocytopenia showed a potential correlation with fluoxetine (2.11) and paroxetine (2.68). AKI risk may be increased with citalopram (1.39), escitalopram (1.36), fluvoxamine (3.24), and COX-2 inhibitors (2.24). DDI signal analysis suggested that citalopram in combination with propionic acid derivatives (additive model = 0.01, multiplicative model = 1.14, and CRR = 3.13) might increase the risk of bleeding. Paroxetine combined with NSAIDs (additive model = 0.014, multiplicative model = 2.65, and CRR = 2.99) could potentially increase the risk of thrombocytopenia. Sertraline combined with NSAIDs (Ω_025_ = 0.94, multiplicative model = 2.14) might be associated with an increasing risk of AKI. Citalopram combined with propionic acid derivatives (Ω_025_ = 1.08, multiplicative model = 2.17, and CRR = 2.42) could be associated with an increased risk of acute kidney injury. **Conclusions**: Certain combinations of SSRIs and NSAIDs might further elevate these risks of gastrointestinal bleeding, thrombocytopenia, and acute kidney injury in patients with depression. Given the potential drug–drug interactions, heightened clinical vigilance is advised when prescribing SSRIs and NSAIDs in combination to patients with depression.

## 1. Introduction

Depression is a widespread mental disorder, affecting approximately 280 million individuals globally, according to the World Health Organization [1]. Depression is the third leading cause of disability worldwide [2] and is characterized by significant and persistent loss of interest or depressed mood, accompanied by cognitive, behavioral, and physical disorders [3]. Currently, a range of antidepressant medications and psychotherapeutic approaches have been validated as effective treatments for depression [4,5]. In pharmacotherapy, selective serotonin reuptake inhibitors (SSRIs) are frequently prescribed as first-line antidepressants due to their proven efficacy and favorable safety profile [6].

Studies have shown that systemic inflammation is associated with an increased risk of depression [7,8]. Multiple meta-analyses of randomized controlled trials (RCTs) have revealed that anti-inflammatory drugs exhibit antidepressant effects and can amplify the effectiveness of conventional antidepressant therapies [9,10,11]. Therefore, non-steroidal anti-inflammatory drugs (NSAIDs) are frequently used alongside antidepressants as adjunctive therapy for depression. However, evidence also suggests that the combination of NSAIDs and SSRIs may elevate the risk of abnormal bleeding, including upper gastrointestinal bleeding [12] and intracranial bleeding [13].

The co-administration of SSRIs and NSAIDs is prevalent in the pharmacological treatment of depression, yet clinical evidence and safety trials on their combined use remain scarce, with varying efficacy and safety profiles across different SSRIs and NSAIDs. Hence, it is crucial to conduct a comprehensive analysis of the potential risks of adverse drug reactions in depressed patients concurrently using SSRIs and NSAIDs, leveraging large-scale post-marketing pharmacovigilance data.

The U.S. Food and Drug Administration (FDA) Adverse Event Reporting System (FAERS) is a spontaneous reporting system that gathers reports of drug use and adverse events (AEs) [14]. It includes AE reports voluntarily submitted by physicians, pharmacists, other healthcare professionals, manufacturers, and consumers from both the U.S. and abroad.

This study performed data mining on adverse event reports related to depressed patients using SSRIs and NSAIDs within the FAERS database, with the aim of elucidating the real-world characteristics of such patients and identifying potential adverse event signals. Through this research, we aim to provide preliminary data that may offer clinicians useful monitoring considerations regarding the safety of SSRIs and NSAIDs in combination.

## 2. Results

### 2.1. Results of Research Subject Selection

**Figure 1:**
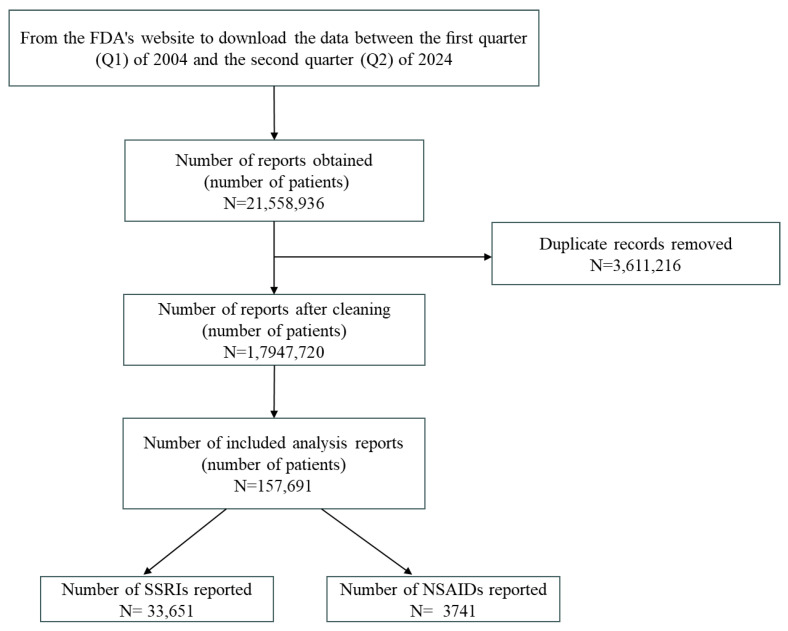
Flowchart of inclusion and screening of study objects.

Data from the first quarter of 2004 through the second quarter of 2024 were retrieved from the FDA website, yielding a total of 21,558,936 reported cases. After excluding duplicate reports, the number of distinct patients was 17,947,720. Depressed patients were identified through MeDRA terminology, with a total of 157,691 individuals included in the analysis. Of these, 33,651 reported the use of SSRIs, whereas 3741 indicated the use of NSAIDs. The inclusion process for the study population is depicted in Figure 1.

### 2.2. Basic Characteristics of the Research Subjects

As presented in Table 1, the total sample size for the analysis was 157,691 cases, including 121,449 in the control group (no target drug), 32,501 in the SSRI-only group, and 2591 in the NSAID-only group. In addition, 1150 patients were included in a group that used both SSRIs and NSAIDs. The SSRI-only group exhibited the youngest mean age at 47.01 years (SD = 23.02), whereas the NSAID-only group had the oldest mean age at 49.85 years (SD = 17.46). The majority of reports across different groups were female, with only a small proportion of cases reporting unknown gender. In the control group, most reports were from 2014 to 2018 (29.8%). In the SSRI-only group, most reports came from 2019 to 2024 (39.7%). The NSAID-only group primarily reported from 2014 to 2018 (31.7%); the same was true for the SSRI and NSAID combination groups, with most reports dating from 2014 to 2018 (37.4%). In the control group, 62.7% of the reports originated from the United States. The SSRI-only group had the largest proportion of reports from the United Kingdom (29.8%). In the NSAID-only group, most reports came from the U.S. (64.7%). In the combined SSRI and NSAID group, most reports were from the United Kingdom (45.4%).

### 2.3. Frequency of Use of SSRI and NSAIDs in AE Reports in Patients with Depression

**Figure 2:**
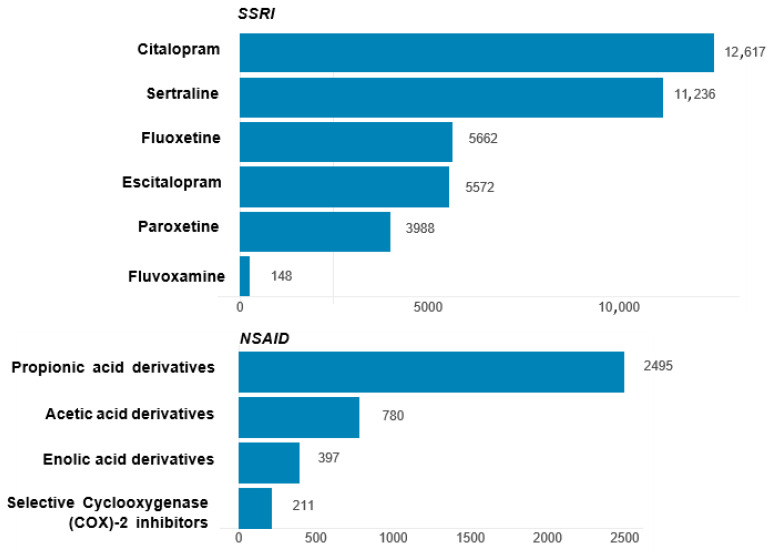
Frequency of AE reports using SSRIs and NSAIDs in patients with depression.

As depicted in Figure 2, of the various SSRIs, citalopram and sertraline were the most prevalent, with citalopram leading at 12,617 (37.49%) reported cases, followed by sertraline at 11,236 (33.39%). Fluvoxamine was the least utilized, with only 148 (0.44%) cases. Among NSAIDs, propionic acid derivatives dominated, and the number of reported cases was as high as 2495 (65.73%).

### 2.4. Signal Detection for Various SSRIs and NSAIDs and AEs of Interest

We studied the effect of the use of individual SSRIs or individual NSAIDs on depressed patients (Table 2). The results suggested potential signals that citalopram, escitalopram, paroxetine, fluvoxamine, and sertraline might be associated with an increased risk of gastrointestinal bleeding, with cROR of 2.81 (95%CI 2.30–3.44), 2.27 (95%CI 1.67–3.06), 2.17 (95%CI 1.52–3.10), 3.58 (95%CI 0.88–14.47), and 1.69 (95%CI 1.31–2.17), respectively. Among NSAIDs, propionic acid derivatives [cROR = 3.17, 95%CI (2.18–4.61)] may increase the risk of gastrointestinal bleeding.

Fluoxetine [cROR = 2.11, 95%CI (1.60–2.78)] and paroxetine [cROR = 2.68, 95%CI (2.01–3.59)] showed a strong risk signal association with thrombocytopenia, whereas the NSAIDs did not produce a risk signal associated with thrombocytopenia.

Among SSRIs, citalopram, escitalopram, and fluvoxamine all showed potential associations with an increased risk of acute kidney injury, with a cROR of 1.39 (95%CI 1.20–1.60), 1.36 (95%CI 1.10–1.67), and 3.24 (95%CI 1.43–7.34). Fluvoxamine showed the strongest signal of potential association with an increased risk of acute kidney injury. Selective COX-2 inhibitors in NSAIDs [cROR = 2.24, 95%CI (1.00–5.06)] also appeared to be associated with an increased risk of acute kidney injury. The cROR value of sertraline was 0.80, and the lower bound of the 95%CI confidence interval was less than 1, suggesting no significant association with AKI than other drugs.

### 2.5. Signal Detection for Various SSRI-NSAID Combinations and AEs of Interest

Four statistical models were used to assess the possible association between various drug combinations and targeted adverse reactions: the Ω shrinkage model, the additive model, the multiplicative model, and the combination risk ratio (CRR) model. The results of the analysis are shown in Table 3.

Gastrointestinal bleeding: In the Ω shrinkage model, no statistically significant signal was observed between the combination of citalopram and NSAID1 (propionic acid derivative) and bleeding [Ω_025_ = 0.89, 95%CI (−0.11, 1.89)]. In the additive model, the effect size was estimated to be 0.01, indicating a potential weak association between the drug combination and gastrointestinal bleeding. The multiplicative model yielded a value of 1.14, indicating a modest potential risk increase. The CRR model showed a more pronounced signal with 3.13, exceeding the threshold of 2. Taken together, although the signal remains insignificant in the Ω shrinkage model and results vary across models, there is a reasonable concern that the combination of citalopram and propionic acid derivatives may elevate the risk of bleeding.

Thrombocytopenia: The Ω shrinkage model for paroxetine combined with NSAID showed a value of 1.17, with a 95%CI of (−0.46, 2.81), indicating no statistically significant signal. The additive model estimated an effect size of 0.014, suggesting a potential increase in risk. However, the multiplicative and CRR models yielded values of 2.65 and 2.99, respectively, indicating a potentially significant increase in thrombocytopenia risk with this combination. Although the Ω shrinkage model did not reveal a significant signal, the results from the other models warrant further scrutiny of the potential for the paroxetine and NSAID combination to elevate the risk of thrombocytopenia.

Acute kidney injury: The additive and CRR models, with values of −0.01 and 1.62 respectively, showed no significant signal between sertraline and NSAID use. However, the Ω shrinkage model [Ω_025_ = 0.94, 95%CI (0.09, 1.80)] and the multiplicative model (2.14) suggested that combining sertraline with NSAIDs may be linked to an increased risk of acute kidney injury. Although some models did not reveal a significant signal, the results from others indicate a potential association that warrants further investigation to confirm this risk.

Although the additive model did not yield a statistically significant result (−0.003), the Ω shrinkage model [Ω_025_ = 1.08, 95%CI: 0.23–1.93], the additive model (2.17), and the CRR model (2.42) all indicated potential risk signals for acute kidney injury with the combination of citalopram and propionic acid derivatives. These findings suggest that this combination may be associated with an elevated risk of acute kidney injury.

## 3. Discussion

In this study, we not only identified adverse reaction signals for SSRIs and NSAIDs when used individually through proportional imbalance analysis but also applied four statistical models to evaluate the adverse reaction signals of various drug combinations. Our findings indicate that certain combinations of SSRIs and NSAIDs may elevate the risk of gastrointestinal bleeding, thrombocytopenia, and acute kidney injury in patients with depression, underscoring the importance of careful consideration when prescribing these combinations.

The combination of SSRIs and NSAIDs may increase the risk of gastrointestinal bleeding, which is consistent with the conclusions of previous studies. SSRIs alone have been shown to increase the risk of gastrointestinal bleeding, and this class of drugs is thought to be able to block platelet reuptake of 5-hydroxytryptamine (5-HT), thereby impairing the function that leads to hemostasis [15]. NSAIDs exert their anti-inflammatory effects mainly by inhibiting the activity of cyclooxygenase (COX-1 and COX-2) [16]. Due to COX-1 inhibition, prostaglandin (PGE_2_) is reduced, leading to gastrointestinal problems associated with gastrointestinal mucosal damage, such as gastric ulcers, perforation, and bleeding. This is one of the main side effects of NSAIDs [17]. Our study found that the combination of SSRIs and NSAIDs, especially citalopram and propionic acid derivatives, increased the risk of gastrointestinal bleeding. Therefore, clinicians should maintain heightened vigilance when considering this combination therapy, with particular attention to potential gastrointestinal bleeding.

In addition, this study found that the combination of Paxil (paroxetine) and NSAIDs increased the potential risk of thrombocytopenia. Paroxetine, as an SSRI, can effectively inhibit platelet uptake of 5-HT, leading to decreased platelet aggregation function [18]. At the same time, a key function of COX-1 under normal physiological conditions is to participate in platelet aggregation. When COX-1 is inhibited, platelet aggregation is also inhibited [19]. Therefore, when Paxil is combined with NSAIDs, the inhibition of platelet aggregation by both drugs is compounded. This makes it harder for platelets to aggregate effectively to form clots to stop bleeding when blood vessels are damaged, increasing the risk of bleeding. Prolonged or severe bleeding may lead to excessive depletion of platelets, which indirectly increases the risk of thrombocytopenia.

The combination of SSRIs and NSAIDs may also increase the risk of acute kidney injury, especially when sertraline is used in combination with an NSAID or citalopram is used in combination with propionic acid derivatives. NSAIDs have been shown to be a common cause of acute kidney injury [20,21], but there is no direct evidence of an association between SSRIs and acute kidney injury. Our research found that the citalopram, escitalopram, and fluvoxamine in SSRIs showed risk signals of AKI. A recent large-scale pharmacovigilance study revealed high AKI occurrences for specific SSRIs—escitalopram (265 cases) and citalopram (264 cases)—among antidepressants [22]. These findings are consistent with our observations, further supporting the potential risk of acute kidney injury associated with SSRIs. Although the exact mechanisms remain incompletely understood, it is noteworthy that SSRI-associated AKI may likely be secondary to rhabdomyolysis. Published case reports implicate escitalopram [23] and fluvoxamine [24] in the development of rhabdomyolysis, a severe condition characterized by muscle breakdown and myoglobin release [25]. Rhabdomyolysis is a well-established common cause of AKI [26], occurring in nearly 50% of patients [27]. Therefore, it is reasonable to think that AKI may be caused by SSRI-induced rhabdomyolysis. When SSRIs are used in combination with NSAIDs, there may be an increased risk of AKI because both may affect kidney function.

In clinical treatment, to improve the therapeutic effect, it is usually necessary to apply two or more drugs, that is, a combination of drugs. However, multidrug combinations present new problems. Specifically, not only can a single drug cause adverse events, but there may be an increased risk of adverse events due to drug–drug interactions (DDI) [28]. Therefore, the safety monitoring of DDI to ensure the safety and efficacy of drug therapy is of paramount importance. Due to the limited study population in clinical trials and the frequent exclusion of patients receiving combination therapy, the various adverse effects of drug combinations are difficult to identify in a timely manner before the drug is marketed [29]. We have addressed this gap using the FAERS database, which gathers real-world data on adverse events associated with various drugs, enabling the evaluation of combination drug safety and offering valuable insights for clinical practice.

This study has several limitations.

First, as with all pharmacovigilance studies using spontaneous reporting systems [30], our analysis of FAERS data inherits well-known limitations, including underreporting and selective reporting [31]. The absence of denominator data precludes the calculation of true incidence rates or population-attributable risks. Important confounding factors including patient demographics (age, sex), comorbidities, and treatment durations could not be adequately controlled for in this analysis [32]. In addition, the reliance on a single pharmacovigilance database (FAERS) may introduce biases inherent to its population coverage [33,34]. To strengthen the reliability of detected signals, future research should implement multi-database validation approaches.

Second, the low frequency of some adverse event reports raises questions about the applicability of certain statistical models, which require further investigation. While we employed multiple statistical approaches to mitigate this limitation, the fundamental challenges of sparse data persist. From a methodological perspective, future studies could significantly enhance signal detection capabilities by integrating advanced data mining techniques, including association rule mining [35,36] and machine learning algorithms [37].

Third, while we employed multiple analytical approaches (disproportionality analysis; Ω shrinkage; and additive, multiplicative, and CRR models), these methods can only detect statistical signals rather than establish causal relationships [38,39]. Therefore, for the detected adverse reaction signals, confirmatory studies should still be conducted in the future, such as physiological mechanism studies and prospective epidemiological designs, to verify their associations. Taking the AKI signal we detected as an example, despite the limited number of reports on this adverse drug reaction associated with SSRIs, several case reports support our hypothesis that SSRIs may induce rhabdomyolysis [23,24,40], which could subsequently contribute to AKI development through secondary mechanisms. However, this proposed pathway remains speculative given the FAERS database’s inherent limitations in capturing detailed biomarker data. It is extremely necessary to carry out mechanistic studies to elucidate the precise pathophysiological pathways and large-scale population-based epidemiological investigations to validate the observed signal and quantify its clinical significance. Only through such rigorous validation in the future can we determine whether the detected signals represent some true associations worthy of clinical consideration. Therefore, despite the inherent limitations of spontaneous reporting systems, this study demonstrates the value of multi-model signal detection in pharmacovigilance. By identifying potential risk signals, it provides a foundation for further investigation while underscoring the importance of early warning systems in drug safety monitoring.

## 4. Materials and Methods

### 4.1. Data Source

The FAERS data, which are publicly available, can be downloaded from the FDA website https://www.fda.gov/ (accessed on 3 May 2024). The FAERS database is made up of seven sub-datasets: demographics (DEMO), reactions (REAC), drugs (DRUG), indications (INDI), outcomes (OUTC), therapy start and end dates (THER), and report sources (RPSR). The ‘DEMO’ table includes basic information such as gender, age, AE date, and the country of occurrence. The ‘DRUG’ table provides details on drug name, administration route, and dosage. The ‘REAC’ table lists adverse event names, and the ‘OUTC’ table details patient outcomes. The ‘RPSR’ table contains information on the report source, the ‘INDI’ table includes patient indications, and the ‘THER’ table provides data on therapy start and end dates.

**Figure 3:**
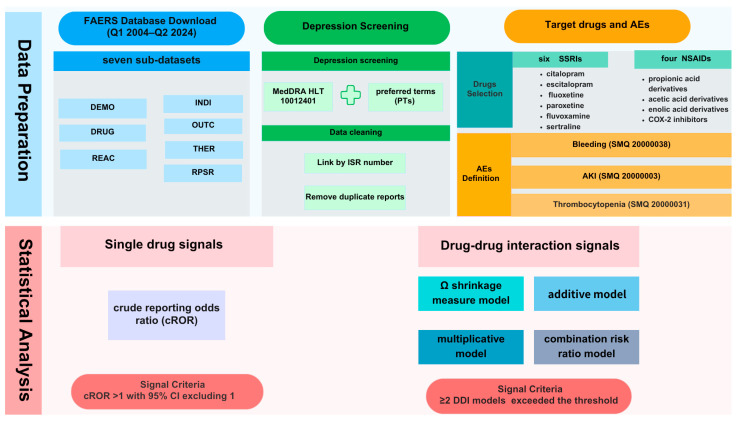
FAERS data analysis process.

The FAERS database for this study included data reported from the first quarter of 2004 through the second quarter of 2024. The key process of the analysis is shown in Figure 3.

### 4.2. Depression Screening and Data Cleaning

According to the Medical Dictionary for Regulatory Activities (MedDRA), the high-level term (HLT) for “Depressive Disorders” is 10012401, which is encoded by the corresponding preferred terms (PTs) 10077804, 10071324, 10067371, 10080836, 1001496, 10068631, 10078366, 10012378, 10057840, 10012397, and 10070606, which were used to screen for depressed patients.

The unique ISR number links all relevant data across the different sub-datasets. Due to the presence of duplicate reports in the FAERS database, we removed the older entries by sorting the case identification numbers.

### 4.3. Selection of Target Adverse Events

This study focused on three adverse reaction events: bleeding, thrombocytopenia, and acute kidney injury (AKI). Both SSRIs [41] and NSAIDs [42] are independently associated with an increased risk of bleeding, and their combination further exacerbates this risk [43]. Thrombocytopenia is reported to be potentially associated with specific SSRIs (e.g., citalopram [44]) and NSAIDs (e.g., propionic acid-derived NSAIDs [45]). NSAIDs has been found to be the major cause of drug-induced AKI [46]. These adverse events can lead to significant clinical consequences, including hospitalization and long-term morbidity.

### 4.4. Target Drugs and Definition of AEs

All reports involving depressed patients treated with SSRIs or NSAIDs were included in the analysis, regardless of whether the drug was identified as the primary or secondary suspect (“PS” or “SS”), concomitant (“C”), or involved in an interaction (“I”) in causing the adverse event.

The SSRIs most commonly used to treat depression are citalopram, escitalopram, fluoxetine, paroxetine, fluvoxamine, and sertraline; consequently, our analysis centered on these six medications [47,48]. In addition, we selected the most widely used NSAIDs, which were categorized as follows: propionic acid derivatives (ibuprofen, naproxen, ketoprofen, flurbiprofen, loxoprofen, and oxaprozin), acetic acid derivatives (diclofenac, indomethacin, ketorolac, sulindac, etodolac, nabumetone, and tolmetin), enolic acid derivatives (oxicams, meloxicam, piroxicam, lornoxicam, and tenoxicam), and selective cyclooxygenase (COX)-2 inhibitors (celecoxib and rofecoxib). Aspirin was excluded from the study due to its widespread use for disease prevention.

AEs in FAERS are coded using the preferred terms (PTs). Standardized MedDRA Queries (SMQs) are groupings of MedDRA terms, typically at the PT level, associated with adverse events.

We extracted AE reports using PTs outlined in MedDRA version 22.0. Acute kidney injury (AKI) was identified using PTs under SMQ code 20000003. Thrombocytopenia was defined by SMQ code 20000031. For bleeding, we employed six PTs from the SMQ ‘Hemorrhages’ (SMQ code: 20000038), including the following PTs: duodenal ulcer with bleeding (PT code: 10013839), hemorrhagic duodenitis (PT code: 10013865), gastric ulcer with bleeding (PT code: 10017826), hemorrhagic gastritis (PT code: 10017866), gastrointestinal hemorrhage (PT code: 10017955), and fecal hemoglobin (PT code: 10018836).

### 4.5. Statistical Analysis

The impact of taking SSRIs or NSAIDs individually on the selected adverse events was analyzed using the crude reporting odds ratio (cROR) and 95% confidence intervals (95% CI), which are commonly used indicators for detecting adverse event signals. A cROR greater than 1, with a 95%CI excluding 1, was considered the threshold for statistical signal detection [49]. The higher the cROR value, the stronger was the signal of the adverse reaction. A two-by-two contingency table served as the analytical framework (Table 4).

cROR=(a/b)/(c/d)

$$95\%CI=e^{ln(ROR)\pm 1.96\sqrt{\left(\frac{1}{a}+\frac{1}{b}+\frac{1}{c}+\frac{1}{d}\right)}}$$

Four criteria were employed to evaluate drug–drug interaction (DDI) signals: (1) the Ω shrinkage measure model, (2) the additive model, (3) the multiplicative model, and (4) the combination risk ratio (CRR) model [50]. The 4 × 2 contingency table served as the analytical framework (Table 5). In this study, if the results from at least two of the four models exceeded the threshold, the drug combination was considered a positive signal for DDI-related adverse events. Statistical analysis was conducted using R version 4.3.3.

(1) Ω shrinkage measure model:
  $$\;\Omega_{025}=\Omega -\frac{\phi (0.975)}{log(2)\sqrt{n_{111}}}>0$$
(2) Additive model:
  $$\frac{n_{111}}{n_{11+}}-\frac{n_{101}}{n_{10+}}-\frac{n_{011}}{n_{01+}}-\frac{n_{001}}{n_{00+}}>0$$
(3) Multiplicative model:
  $$\frac{n_{111}\times n_{001}\times n_{10+}\times n_{01+}}{n_{11+}\times n_{00+}\times n_{101}\times n_{011}}>1$$
(4) CRR model:
  CRR=PRRdrug SSRIs ∩NSAIDsdrugmax(PRRdrug SSRIs, PRRdrug NSAIDs)>2
  $$n_{111}\geq 3,\;{PRR}_{\mathrm{drug}\;\mathrm{SSRIs}\;\cap \;\mathrm{drug}\;\mathrm{NSAIDs}\;}>2,\;\chi_{\;\mathrm{drug}\;\mathrm{SSRIs}\;\cap \;\mathrm{drug}\;\mathrm{NSAIDs}\;}^{2}>4$$

## 5. Conclusions

Most SSRIs (citalopram, escitalopram, paroxetine, fluvoxamine, and sertraline) and propionic acid derivatives (non-steroidal anti-inflammatory drugs) appear to potentially be linked to gastrointestinal bleeding when used alone. The combination of citalopram and propionic acid derivatives showed signals suggesting a high potential increased risk of bleeding. Fluoxetine, when used alone, may potentially raise the risk of thrombocytopenia. Paroxetine, in combination with NSAIDs, could possibly elevate the risk of thrombocytopenia. Citalopram, escitalopram, fluvoxamine, and selective COX-2 inhibitors alone demonstrated potential associations with an increased risk of acute kidney injury. Moreover, combinations of sertraline with NSAIDs and citalopram with propionic acid derivatives also showed possible links to acute kidney injury.

## Figures and Tables

**Table 1:** Basic characteristics of the population grouped according to reported drug use combinations.

		Control	Only SSRIs	Only NSAIDs	SSRIs + NSAIDs
n		121,449	32,501	2591	1150
Age, (mean (SD))		47.91 (19.77)	47.01 (23.02)	49.85 (17.46)	47.57 (20.53)
Age, n (%)	<18	6014 (5.0)	2502 (7.7)	77 (3.0)	67 (5.8)
	18–45	29,284 (24.1)	9784 (30.1)	626 (24.2)	349 (30.3)
	46–64	32,700 (26.9)	6981 (21.5)	864 (33.3)	344 (29.9)
	>65	17,711 (14.6)	6625 (20.4)	376 (14.5)	204 (17.7)
	Unknown	35,740 (29.4)	6609 (20.3)	648 (25.0)	186 (16.2)
Sex, n (%)	Female	78,358 (68.1)	19,323 (65.2)	1711 (68.5)	738 (70.6)
	Male	36,418 (31.6)	10,193 (34.4)	780 (31.2)	302 (28.9)
	Unknown	303 (0.3)	107 (0.4)	6 (0.2)	5 (0.5)
Year, n (%)	2004–2008	25,546 (21.0)	3821 (11.8)	443 (17.1)	137 (11.9)
	2009–2013	28,356 (23.3)	5916 (18.2)	698 (26.9)	232 (20.2)
	2014–2018	36,173 (29.8)	9877 (30.4)	822 (31.7)	351 (30.5)
	2019–2024	31,374 (25.8)	12,887 (39.7)	628 (24.2)	430 (37.4)
Country, n (%)	United States	76,135 (62.7)	6132 (18.9)	1677 (64.7)	158 (13.7)
	United Kingdom	5719 (4.7)	9692 (29.8)	201 (7.8)	522 (45.4)
	France	5631 (4.6)	3494 (10.8)	46 (1.8)	67 (5.8)
	Germany	3927 (3.2)	2309 (7.1)	105 (4.1)	78 (6.8)
	Others	20,895 (17.2)	9573 (29.5)	392 (15.1)	271 (23.6)
	Not Specified	9142 (7.5)	1301 (4.0)	170 (6.6)	54 (4.7)

**Table 2:** Signal detection for various SSRIs and NSAIDs and targeted AEs.

Drugs		Bleeding	Thrombocytopenia	Acute Kidney Injury
SSRI				
	citalopram	2.81 [2.30, 3.44]	1.12 [0.87, 1.43]	1.39 [1.20, 1.60]
	escitalopram	2.27 [1.67, 3.06]	1.09 [0.75, 1.58]	1.36 [1.10, 1.67]
	fluoxetine	1.36 [0.93, 1.97]	2.11 [1.60, 2.78]	1.16 [0.93, 1.44]
	paroxetine	2.17 [1.52, 3.10]	2.68 [2.01, 3.59]	1.26 [0.98, 1.61]
	fluvoxamine	3.58 [0.88, 14.47]	2.84 [0.70,11.49]	3.24 [1.43, 7.34]
	sertraline	1.69 [1.31, 2.17]	1.08 [0.83, 1.41]	0.80 [0.66, 0.97]
NSAID				
	propionic acid derivatives	3.17 [2.18, 4.61]	1.34 [0.82, 2.21]	1.32 [0.97, 1.79]
	acetic acid derivatives	1.69 [0.70, 4.08]	0.53 [0.13, 2.13]	1.30 [0.75, 2.25]
	enolic acid derivatives	1.99 [0.64, 6.21]	0.52 [0.07, 3.72]	0.78 [0.29, 2.09]
	selective cyclooxygenase (COX)-2 inhibitors	1.24 [0.17, 8.87]	2.99 [0.96, 9.38]	2.24 [1.00, 5.06]

**Table 3:** Signal detection for various drug combinations and targeted AEs.

DDI Combination	n_111_	n_11+_	Ω Shrinkage Model	Additive Model	Multiplicative Model	CRR Model
DDI for Bleeding						
SSRI + NSAID	19	1150	0.49 (−0.16, 1.34)	−0.001	0.87	2.25
Citalopram + NSAID	9	415	0.56 (−0.39, 1.50)	0	0.9	2.43
Fluoxetine + NSAID	3	229	0.20 (−1.43, 1.83)	−0.005	0.99	1.31
Sertraline + NSAID	5	417	−0.03 (−1.30, 1.23)	−0.001	0.71	1.21
Citalopram + NSAID1	8	271	0.89 (−0.11, 1.89)	0.01	1.14	3.13
Sertraline + NSAID1	4	322	−0.16 (−1.58, 1.25)	−0.01	0.63	1.06
DDI for Thrombocytopenia						
SSRI+NSAID	12	1150	0.68 (−0.13, 1.50)	−0.004	1.71	1.55
Citalopram + NSAID	3	415	0.18 (−1.45, 1.82)	−0.008	1.14	1.26
Fluoxetine + NSAID	3	229	0.28 (−1.35, 1.92)	−0.006	1.17	1.37
Paroxetine + NSAID	3	85	1.17 (−0.46, 2.81)	0.014	2.65	2.99
Sertraline + NSAID	3	417	0.21 (−1.42, 1.85)	−0.008	1.18	1.26
Citalopram + NSAID1	3	271	0.67 (−0.96, 2.30)	−0.001	1.73	1.89
Fluoxetine + NSAID1	3	160	0.69 (−0.94, 2.33)	−0.001	1.64	1.98
Sertraline + NSAID1	3	326	0.46 (−1.17, 2.09)	−0.01	1.45	1.54
DDI for Acute Kidney Injury						
SSRI+NSAID	27	1150	0.50 (−0.05, 1.04)	−0.02	1.39	1.6
Citalopram + NSAID	13	415	0.62 (−0.16, 1.40)	−0.11	13.35	1.86
Escitalopram + NSAID	4	150	0.29 (−1.13, 1.70)	−0.02	1.17	1.57
Sertraline + NSAID	11	417	0.94 (0.09, 1.80)	−0.01	2.14	1.62
Citalopram + NSAID1	11	271	1.08 (0.23, 1.93)	−0.003	2.17	2.42
Sertraline + NSAID1	6	326	0.37 (−0.78, 1.53)	−0.021	1.41	1.11

**Table 4:** 2 × 2 contingency table for crude reporting odds ratio calculation.

	With an Adverse Event of Interest	Without an Adverse Event of Interest
With a drug of interest	a	b
Without a drug of interest	c	d

**Table 5:** 4 × 2 contingency tables for drug–drug interaction signal analysis.

	AEs of Interest	Other AEs	Total
Concomitant use of SSRIs and NSAIDs	n_111_	n_110_	n_11+_
SSRIs without NSAIDs	n_101_	n_100_	n_10+_
NSAIDs without SSRIs	n_011_	n_010_	n_01+_
Neither SSRIs nor NSAIDs	n_001_	n_000_	n_00+_
Total	n_++1_	n_++0_	n_+++_

## Data Availability

The raw FAERS data supporting this study are publicly available on the FDA website https://www.fda.gov/drugs/drug-approvals-and-databases/fda-adverse-event-reporting-system-faers (accessed on 3 May 2024). Processed datasets (.xlsx format) used for statistical analyses has been provided as Supplementary Material. Further inquiries can be directed to the corresponding author.

## Supplementary Materials

The following supporting information can be downloaded at: https://www.mdpi.com/article/10.3390/ph18071062/s1.
